# Non-destructive determination of ethanol levels in fermented alcoholic beverages using Fourier transform mid-infrared spectroscopy

**DOI:** 10.1186/s13065-017-0257-5

**Published:** 2017-03-24

**Authors:** Ayalew Debebe, Mesfin Redi-Abshiro, Bhagwan Singh Chandravanshi

**Affiliations:** 1 Department of Chemistry, Addis Ababa University, P.O. Box 1176, Addis Ababa, Ethiopia; 2 Department of Chemistry, Haramaya University, P.O. Box 138, Dire Dawa, Ethiopia

**Keywords:** Ethanol, Fermented alcoholic beverages, Traditional beverages, MIR-PLS, GC-FID, Ethiopia

## Abstract

**Background:**

Traditional fermented alcoholic beverages are indigenous to a particular area and are prepared by the local people using an age-old techniques and locally available raw materials. The main objective of this work was the direct determination of ethanol in traditional fermented alcoholic beverages using mid infrared spectroscopy with partial least squares regression, verifying the robustness of the calibration models and to assess the quality of beverages.

**Results:**

The level of ethanol determination in Ethiopian traditional fermented alcoholic beverages was done using mid infrared spectroscopy with partial least squares regression (MIR-PLS). The calibration and validation sets, and real samples spectra were collected with 32 scans from 850–1200 cm^−1^. A total of 25 synthetic standards (calibration and validation sets) with ethanol (2–10% w/w) and sugars (glucose, fructose, sucrose and maltose) (0–5% w/w) compositions were used to construct and validate the models. Twenty-five different calibration models were validated by cross-validation approach with 25 left out standards. A large number of pre-treatments were verified, but the best pre-treatment was subtracting minimum + 2nd derivative. The model was found to have the highest coefficients of determination for calibration and cross-validation (0.999, 0.999) and root mean square error of prediction [0.1% (w/w)]. For practical relevance, the MIR-PLS predicted values were compared against the values determined by gas chromatography. The predicted values of the model were found to be in excellent agreement with gas chromatographic measurements. In addition, recovery test was conducted with spiking 2.4–6.4% (w/w) ethanol. Based on the obtained recovery percentage, 85.4–107% (w/w), the matrix effects of the samples were not considerable.

**Conclusion:**

The proposed technique, MIR-PLS at 1200–850 cm^−1^ spectral region was found appropriate to quantify ethanol in fermented alcoholic beverages. Among the studied beverages (*Tella*, *Netch Tella*, *Filter Tella*, *Korefe*, *Keribo*, *Borde* and *Birz*), the average ethanol contents ranged from 0.77–9.1% (v/v). *Tej* was found to have the highest ethanol content whereas *Keribo* had the least ethanol content. The developed method was simple, fast, precise and accurate. Moreover, no sample preparation was required at all. However, it should be noted that the present procedure is probably not usable for regulatory purposes (e.g. controlling labelling).

## Background

Fermented alcoholic beverages are the oldest alcoholic drinks of low alcoholic contents [1, 2]. Fermented alcoholic beverages such as beers and wines are complex mixtures mainly composed of ethanol, water and carbohydrates [3–7], and a large number of minor compounds such as alcohols, acids, esters, aldehydes, polyphenols, metals, and amino acids [8–14]. Fermented alcoholic beverages are produced traditionally at small scale as well as industrially at large scale. Traditional fermented beverages are indigenous to a particular area and have been prepared by the local people using an age-old techniques and locally available raw materials [15, 16]; accordingly, different countries have various indigenous fermented alcoholic beverages [17–22].

In Ethiopia many traditional fermented beverages are known. They are high alcoholic beers such as *Tella*, *Korefe*; low alcoholic beers such as *Keribo*, *Buqri*, *Shameta*, *Borde* and wine such as *Tej* made from honey [23, 24]. *Tella*, *Filter Tella* and *Korefe* are made from a mixture of *enkuro* (dark brown toasted flour of barley, maize or sorghum), *bikil* (germinated grain), *gesho* (*Rhamnus prenoides*) and water [23–25]. *Netch Tella* is prepared by a former mixture except using *kita* (5–10 mm thick, pancake-like bread) in place of *enkuro* [23–25]. All *Tella* types are liquid, whereas *Korefe* is semi-liquid. *Tej* is made from water, honey (or sugar in the cruder blends), and crushed *gesho* (*Rhamnus prinoides*) leaves as a fermenting agent [23–26]. *Borde* is a common meal replacer in southern Ethiopia and is prepared from unmalted cereals and their malt [23]. *Keribo* is prepared from deeply roasted barley that is added to boiling water and sugar [23].

For a long time pycnometric determination of the density was the approved reference method to determine the alcoholic strength in spirits and wines. But this method has to be preceded by a distillation step. Electronic densimetry was introduced later on into the determination of alcoholic strength. Similar or better performance was achieved using this method in terms of accuracy and precision [21, 27–29]. All these procedures share the common element that they are inexpensive, and do not require standards, reagents and chemicals. They also mostly do not need sample preparation. However, the densimetric methods are relatively time-consuming. Furthermore, special training of personnel is also required to obtained reproducible results.

Several other methods were also developed for the alcohol determination in the beverages including titration methods [30], enzymatic analysis [31], sequential injection analysis [22] as well as liquid or gas chromatographic methods [32–37]. However, these methods did not offer noticeable advantage over the densimetric reference methods. Furthermore they are more complex, labour intensive and time consuming.

To overcome the problems associated with the methods described above, the content of alcohol in the beverages is now a day determined using spectroscopic techniques with faster and simpler method [22, 38–40]. In addition, no sample preparation other than degassing is required in MIR, NIR, UV–Vis and Raman spectroscopies [38]. FT-MIR spectroscopy has several advantages, firstly, it allows the direct analysis of liquid samples without any sample pre-treatment, except sample dilution which makes the method very simple and is user friendly. Secondly, analytes are monitored simultaneously within milliseconds [41]. The progress in the systematic development of analytical methods for the determination of alcohol in the beverages has been well described by Lachenmeier et al. [41].

In mid-infrared spectroscopy, the determination of alcohols mainly ethanol has been reported at different regions from 4000–600 cm^−1^ [7, 17] with/without multivariate techniques. As reported by different scholars [7, 42, 43], in the region ethanol has three particular absorption sites at 3200–2700, 1200–950 and 900–850 cm^−1^ which are not identical in absorption band, sensitivity and interference effect. The determination was done mainly based on the bands due to the fundamental C–O stretching vibrations [39, 40, 44–46]. Since traditional alcoholic beverages are too diversified either with themselves or with others by different aspects, taking representative samples for calibration and validation sets is practically an impossible case. Therefore, preparing a representative samples (synthetic samples) are mandatory. Thus, the innovation point of this research was constructing an efficient model with few samples and then determining ethanol without the need of sample preparation. Therefore, the main objective of this work was the direct determination of ethanol in traditional fermented alcoholic beverages with MIR-PLS, verifying the robustness of the calibration models (synthetic samples), to allow an assessment of whether the accuracy and precision of the method is fit for purpose and to assess the quality of beverages.

## Experimental

### Instrumentation

Fourier transform infrared spectrometer (Spectra 65, Perkin Elmer, UK) with ZnSe window (1 mL capacity sample holder) in ATR mode was used to generate the spectra of standards and real samples. A gas chromatograph with flame ionization detector (GC 1000, Dani, Italy) was used to determine ethanol in the samples. Balance (Adventurer, OHAUS, China) was used to weigh the samples and standard solutions.

### Reagents and chemicals

Ethanol (99.99%, Fisher Scientific, UK), glucose (Laboratory Reagent, Merck Extra Pure, England), fructose (Laboratory Reagent, Pharmacos Ltd, England), sucrose (Analytical Reagent, Guangdong Guanghya Chemical Factory Co. Ltd, China) and maltose (Laboratory Reagent, The British Drug Houses Ltd, Poole-England) were used to prepare synthetic calibration and validation sets. The total number prepared synthetic standards for calibration or validation sets were 25. Based on cross-validation approach, 25 different calibration models were developed with one left out standard in each model. Each developed model was validated with the corresponding left out standard. Accordingly, the total left out standards (validation sets) were 25. The number of real samples analyzed does not have any relation with the number of synthetic standards used for calibration. It should also be noted that using more than 25 sets of standards will be more time consuming and laborious.

The compositions of the synthetic calibration or validation sets were: ethanol (2–10% w/w), glucose (0–5% w/w), fructose (0–5% w/w), sucrose (0–5% w/w) and maltose (0–5% w/w). There was no correlation between the concentrations of the five components in designing the experimental approach. The concentrations of five components were selected based on their contents in the Ethiopian traditional fermented beverages. The amount of sample required for analysis in MIR was 1 mL. For GC-FID standard solutions ranges from 1–50% (w/w) were prepared from 99.99% (v/v) ethanol in 5% n-propanol (internal standard). Since n-propanol is a common alcohol naturally occurring in fermented beverages, but in much lower concentration compared to ethanol, and since it does not overlap with the peak of ethanol, it was used as an internal standard. Distilled-deionized water was used for washing, dilution of samples and preparation of standards.

### Sampling and sample preparation

For this study, eight most popular Ethiopian traditional fermented beverages, *Tej* (honey wine), *Tella* (a malt beverage like beer), *Korefe*, *Keribo*, *Birz*, *Netch Tella*, *Filter Tella* and *Borde* were selected. The samples were collected into two rounds. In one round a total of 57 samples; 15 *Tej*, 15 *Tella*, 6 *Korefe*, 6 *Keribo*, 4 *Birz*, 4 *Netch Tella*, 4 *Filter Tella* and 3 *Borde* were collected randomly from vending houses at different sub-cities of Addis Ababa, the capital city of Ethiopia and from five nearby towns (Sebeta, Dukem, Sululta, Sendafa, and Burayu) of Oromia Regional State. A 500 mL aliquot of each type of the beverages was collected from the three sites of each of the sub-cities of Addis Ababa and nearby towns. A 1000 mL bulk sample was prepared for each sample type from one specific sampling site. This was done by taking 333 mL of the beverage from each of three samples from one place and mixing well in a 1 L volumetric flask. All the samples were collected using glass amber bottles and kept at 4 °C until the analysis time. No sample pre-treatment was made except filtration. These beverages do not contain CO_2_, they are not carbonated, and hence no removal of CO_2_ was required. The samples were not temperated.

### FT^_^MIR analysis

FT-MIR spectra of standards and samples were recorded using Fourier transform infrared spectrometer. Each spectrum was recorded in the region, 1200–850 cm^−1^ with 32 scans. Once more, for each sample the spectra were generated in triplicate. Both air and water backgrounds were used. First air background was used and then water (solvent) background was used. Treatments applied to experimental data and mathematical calibration models were made using Origin Lab-Origin 8 and Math Lab R2009a soft wares.

### Determination of ethanol by GC-FID

After filtration through a 0.45 μm Millipore filter and addition of 5% n-propanol (internal standard), the ethanol content of sample was determined by GC coupled with flame ionization detector (GC-FID). Quantification was based on calibration curve obtained, after injection of samples. The calibration curve was established by a plot of peak area ratio (ethanol: n-propanol) versus concentration  % (w/w); y = 0.13903x + 0.04488, r^2^ = 0.9992. The conversion equation, % (w/w) into % (v/v) was, y = 1.21879x + 0.13712. The calibration curves were developed in triplicates.

The working condition that was used; 3 μL injection volume, initially at 75 °C for 2 min, and then increased to the final temperature of 80 °C in 1 min at rate of 1 °C/min oven temperature, 210 °C injection port temperature, 0.5 bar pressure, 1.4 mL/min flow rate, 300 °C detector temperature and ECTm-5 capillary column.

### Pre-processing and construction of calibration models

For the construction of the multivariate calibration model using partial least squares (PLS), initially all standard spectra were evaluated by principal component analysis (PCA) with the purpose of observing their distribution and the existence of clusters and outliers. Prior to the calibration, the spectral data were pre-processed for optimal performance. The spectra were transformed using different mathematical pre-treatments to remove and minimize the unwanted spectral contribution and to reduce undesirable systematic noise, such as base line variation, light scattering and to enhance the contribution of the chemical composition [47].

The applied data treatment techniques were: subtracting minimum + 1st derivative; subtracting minimum + 2nd derivative + mean centering; subtracting minimum + 1st derivative + mean centering; subtracting minimum + normalization + 1st derivative + mean centering; subtracting minimum + normalization + 2nd derivative + mean centering; subtracting minimum + normalization + mean centering; subtracting minimum + mean centering; subtracting minimum + normalization + 1st derivative; subtracting minimum + normalization + 2nd derivative; subtracting minimum + normalization and subtracting minimum + 2nd derivative.

In constructing the calibration models out of 351 possible latent variables, 6–9 latent variables (PLS components) were utilized by the corresponding models. This is to minimize the error and maximize the prediction capacity of the models.

### Statistical analysis

In order to compare the means of ethanol, one-way ANOVA (significance level α = 0.05) was performed on Origin Lab-Origin 8 software. PLS regression was performed to study the predictive ability of the calibration models. The models were validated using the full cross validation technique, in order to determine the optimal number of latent variables and to detect the outlier samples.

## Results and discussion

### Optimal spectral region selection

Fermented alcoholic beverages are composed of different non-volatile substances such as sugars, proteins, hop, metals, vitamins, colour compounds, etc. [48]. For instance, beer contains 30–40 g/L non-volatile materials. Out of the non-volatile materials found in beer, 80–85% is sugars [48].

**Fig. 1:**
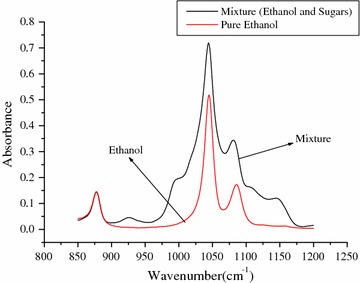
Spectra of pure ethanol and mixture

In addition, in the region 4000–600 cm^−1^, ethanol has an absorbance at 3005–2960, 1200–950 and 900–850 cm^−1^. The absorption is due to C–H stretching, C–O stretching and O–H bending vibration, respectively. Each of them differs by sensitivity and interference effect. However, the spectra at 1200–950 and 900–850 cm^−1^ were the most sensitive and exclusive absorbance region for ethanol, respectively. Thus, the range 1200–850 cm^−1^ was selected as a spectral region, because it satisfied both. Therefore, for quantifying ethanol using PLS at optimal spectral region, ethanol spectra in the presence of sugars were developed (Fig. 1).

### Pre-treatment method selection

Pre-treatment methods are various in numbers and have been applied for different purposes such as for noise reduction, base line correction, etc. [49]. From the data pre-treatment methods which were applied, the best comparative are presented in Table 1. The best model was selected based on the highest coefficients of determination for calibration and cross-validation $( {\text{R}}_{\text{cal}}^{2} ,\;{\text{R}}_{\text{cval}}^{2} ),$ and the smallest standard error of calibration (SEC), standard error of cross validation (SECV) or standard error of prediction (SEP) and the lesser number of latent variables used. Accordingly, based on the data given in Table 1, subtracting minimum + 2nd derivative was the selected data pre-treatment. Though by some extent subtracting minimum and 1st derivative seems more accurate, subtracting minimum and 2nd derivative was selected by the less number of PLS components used for the model and its comparable accuracy with the first one.

**Table 1:** Results of PLS/MIR calibration models for the determination of ethanol in fermented alcoholic beverages

Pre-treatment	PLS component	Calibration		Validation	
		RMSEE	R^2^	RMSEP	R^2^
Raw data	6	0.004	0.999	0.1	0.998
Sub min + 1st derivative	9	0.0001	0.999	0.09	0.999
Sub min + 2nd derivative	6	0.0008	0.999	0.1	0.999
Sub min + mean centering	7	0.002	0.999	0.09	0.999
Sub min + normalization + mean centering	7	0.01	0.999	0.5	0.991
Sub min + normalization + 1st derivative	8	0.004	0.999	0.3	0.989
Sub min + normalization + 2nd derivative	8	0.004	0.999	0.3	0.989

*Sub min* subtracting minimum

### Method validation

Validation of the developed model was done using a validation set that contains 25 synthetic standards. Coefficients of determination for calibration and cross-validation and root mean square error of estimation and prediction are shown in Table 1. The prediction errors of the model (a model with subtracting minimum + 2nd derivative pre-processing) for ethanol contents were 0.1% (w/w). In addition, the predicted amounts was evaluated and compared with the measured values at 99% confidence level. The results obtained indicated that no significance difference between them.

### Comparison of present MIR-PLS with literature reported NIR-PLS and MIR-PLS

Urtubia et al. [50] used NIR to determine ethanol (R^2^ 0.99 and RMSE 1.04 g/L) in wines. Nagarajan et al. [51] applied MIR-PLS to determine ethanol in alcoholic beverages (R^2^ 0.9910, RMSEC 0.2043; R^2^ 0.9896, RMSEV 0.2193). Arzberger and Lachenmeier [52] have applied FT-IR-PLS to determine alcohol content in spirit (R^2^ 0.9937, SECV 0.1996; R^2^ = 0.9859, SEP 0.23) and liquers (R^2^ 0.9993, SECV 0.1995; R^2^ = 0.9855, SEP 0.7472). Kolomiets et al. [53] also applied NIR in alcoholic beverages to determine ethanol (R^2^ 0.984 and RMSE 0.22 g/L). Fu et al. [54] applied SW-NIR-GLSW) to determine alcohol content in wine (R^2^ 0.99, RMSEP 0.55%). Friedel et al. [55] used FT-MIR-PLS at three different operating conditions to determine ethanol in wines (SB-ATR RMSEP 1.49 g/L, transmission-defined pathlength RMSEP 1.02 g/L, transmission-variable pathlength RMSEP 1.52 g/L). Martelo-Vidal and Vázquez [47] applied NIR to predict ethanol in wines (R^2^ 0.991 and RMSEP 1.78 g/L). Shen et al. [56] determined alcohol degree in rice wine using NIR-PLSR (R^2^ 0.972, RMSECV 0.393), MIR-PLSR (R^2^ 0.956, RMSECV 0.494). The prediction accuracy of the present MIR-PLS (R^2^ 0.999 and RMSEP 0.1%, w/w) is comparable to or even better than similar studies in wine, beer and spirit drinks.

### Comparison of MIR-PLS with GC-FID

**Fig. 2:**
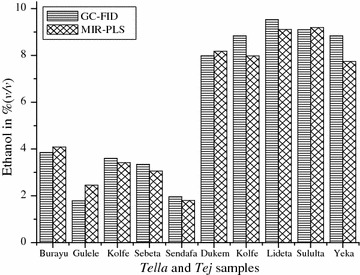
Comparison MIR-PLS and GC-FID on measuring the amount of ethanol in *Tella* and *Tej* samples

The MIR-PLS method was compared with the reference, GC-FID with respect to the obtained ethanol content. At 95% confidence level, the two techniques did not have any significance difference by the ethanol content in % (v/v) (Fig. 2). This indicated that the approach of using synthetically prepared calibration model was efficient to predict the amount of ethanol in different traditional alcoholic beverages. Therefore, for the determination of ethanol in the fermented alcoholic beverages, MIR-PLS was used.

### Recovery test

The accuracy of the developed methods was checked by spiking known concentration of ethanol in the samples. The samples were taken randomly. The selected samples were *Birz*, *Keribo*, *Netch Tella*, *Tej* and *Tella.* The spiked ethanol concentrations and the % recovery ranges are indicated in Table 2.

**Table 2:** Recovery of ethanol in the method

Sample	Unspiked % (w/w)	Spiked 1 % (w/w)	Spiked 2 % (w/w)	Amount added 1 % (w/w)	Amount added 2 % (w/w)	Ethanol % recovery	
						R_1_	R_2_
*Birz*	6.64	9.03	12.0	2.80	5.75	85.4	93.7
*Keribo*	0.42	4.20	5.69	3.79	5.06	99.7	104
*Netch Tella*	2.38	5.58	8.81	3.49	6.36	91.9	101
*Tej*	3.27	6.34	8.45	3.05	4.97	101	104
*Tella*	2.39	4.82	8.57	2.39	5.76	102	107

The recoveries percentages of ethanol for fermented alcoholic beverages were in the range 85.4–107% (w/w) (Table 2). Based on the data obtained since the matrix effects of the samples are not considerable, the proposed technique, MIR-PLS is appropriate to quantify alcohol contents in fermented alcoholic beverages.

### Limit of detection and limit of quantification

The limit of detection and quantification of GC-FID was calculated based on LOD = 3σ of the residues (y-intercepts)/slope and LOD = 10σ of the residues (y-intercepts)/slope, respectively [57]. The obtained limit of detection and qualification were 0.1% (v/v) and 0.4% (v/v), respectively.

### Analysis of samples

Ethanol in the real samples was quantified using MIR-PLS model. Accordingly, the level of ethanol in the samples was determined and the obtained results are illustrated in Table 3.

**Table 3:** The  % (v/v) of ethanol in fermented alcoholic beverage samples using MIR-PLS model

Types of beverages	Number of sample	Ethanol % (v/v)	
		Range	Mean
*Tella*	15	1.3–4.8	2.9 ± 0.3
*Tej*	15	7.6–13	9.1 ± 0.3
*Korefe*	6	4.2–8.2	4.6 ± 0.4
*Keribo*	6	0.4–2.6	0.77 ± 0.3
*Birz*	4	3.8–9.2	6.5 ± 0.8
*Borde*	3	1.0–2.1	2.8 ± 0.4
*Netch Tella*	4	2.7–3.7	3.0 ± 0.3
*Filter Tella*	4	5.2–5.5	7.3 ± 0.4

In Table 3 the average alcoholic contents of the beverages ranged from 0.77–9.1% (v/v). The beverages have significant variations among samples of the same and different types. It might be due to the differences in preparation and fermentation [23, 24, 58], conditions such as temperature, aeration and actions of the micro-organisms [24]. Based on the mean ethanol contents (Table 3) the beverages were in the order: *Keribo* < *Borde* < *Tella* < *Netch Tella* < *Korefe* < *Birz* < *Filter Tella* < *Tej*. Among the studied beverages, *Tej* was found to have highest ethanol content whereas *Keribo* had the least ethanol content. For *Tej* the obtained ethanol concentration, 9.1 ± 0.3% (v/v) was found comparable with the reported one, 11.5% (v/v) with a range of 8.9–13.8% (v/v) [23, 48]. Easily fermentable raw material type (honey or sugar) and a longer fermentation time (5–20 days) allowed *Tej* to be the highest in ethanol content [23, 58]. On the other hand, *Keribo* was found to contain the least ethanol because of shorter fermentation time (overnight). As mentioned by Guranda [59] the ethanol content of *Tella* was 2–4% (v/v), in comparison with this report, the obtained ethanol content, 2.9 ± 0.3% was in the range. As stated by Guranda [59] and Debebe [23] the ethanol content of *Filter Tella* was 5–14.5% (v/v); the obtained value, 7.3 ± 0.4% (v/v) was found comparable and within the reported range. Though both *Filter Tella* and normal *Tella* are *Tella* types, *Filter Tella* was found a head of normal *Tella* in ethanol content. Again, it is due to fermentation time difference.

From the standard deviations which are presented in Table 3, there is no significant scattering of the data. Again, from the obtained recovery percentage (85.4–107% w/w), the matrix effects of the samples were not considerable. This showed that the model has better precision and accuracy in the prediction of ethanol. On the other hand, in the usual trend of multivariate techniques calibration model was developed with a large number of real samples collected from different areas. As a result, since traditional beverages known by non-uniform composition, they require too large number of samples for constructing representative calibration. This is too tedious and time consuming. In contrast, the developed model without using real samples (the usual trained), but using few synthetic standards was found comparable with the reference GC-FID. This showed that the method is simple and fast with no significant sample preparation except filtration.

## Conclusion

Ethanol has three specific spectral regions; 3005–2960, 1200–950 and 900–850 cm^−1^. Among the regions that had the least interfering effect and a comparable data with the GC-FID was 1200–850 cm^−1^. The developed and validated technique at 1200–850 cm^−1^ region allows the direct determination of ethanol in fermented beverages. The proposed MIR-PLS technique at 1200–850 cm^−1^ is found to be an appropriate method for ethanol determinations in fermented beverages. However, it should be noted that the present current procedure is probably not usable for regulatory purposes (e.g. controlling labelling).

## References

[1] Bacha K, Mehari T, Ashenafi M (1999). Microbiology of the fermentation of shamita, a traditional Ethiopian fermented beverages. SINET Ethiop J Sci.

[2] Desta B (1977). A survey of the alcoholic contents of traditional beverages. Ethiop Med J.

[3] Tipparat P, Lapanantnoppakhun S, Jakmunee J, Grudpan K (2001). Determination of ethanol in liquor by near-infrared spectrophotometry with flow injection. Talanta.

[4] Kuria MW, Olando Y (2012). Alcohol dependence: does the composition of the available beverages promote it?. Open J Psychiatry.

[5] Wang M-L, Choong Y-M, Su N-W, Lee M-H (2003). A rapid method for determination of ethanol in alcoholic beverages using capillary gas chromatography. J Food Drug Anal.

[6] Voica C, Dehelean A, Pamula A (2009). Method validation for determination of heavy metals in wine and slightly alcoholic beverages by ICP-MS. J Phys.

[7] Inon FA, Garrigues S, Guardia M (2006). Combination of mid- and near-infrared spectroscopy for the determination of the quality properties of beers. Anal Chim Acta.

[8] Madrera RR, Valles BS (2007). Determination of volatile compounds in cider spirits by gas chromatography with direct injection. J Chromatogr Sci.

[9] Puertolas E, Alvarez I, Raso J (2011). Changes in phenolic compounds of Aragón red wines during alcoholic fermentation. Food Sci Technol Int.

[10] Abernathy DG, Spedding G, Starcher B (2009). Analysis of protein and total usable nitrogen in beer and wine using a microwell ninhydrin assay. J Inst Brew.

[11] Montanari L, Perretti G, Natella F, Guidi A, Fantozzis P (1999). Organic and phenolic acid in beer. Lebensm-Wiss U-Technol.

[12] Melo Coelho NM, Parrilla C, Cervera ML, Pastor A, Guardia M (2003). High performance liquid chromatography—atomic fluorescence spectrometric determination of arsenic species in beer samples. Anal Chim Acta.

[13] Sterckx FL, Saison D, Delvaux FR (2010). Determination of volatile monophenols in beer using acetylation and headspace solid-phase micro-extraction in combination with gas chromatography and mass spectrometry. Anal Chim Acta.

[14] Gorinstein S, Zemser M, Vargas-Albores F, Ochoa J-L, Paredes-Lopez O, Scheler C, Salnikow J, Martin-Belloso O, Trakhtenberg S (1999). Proteins and amino acids in beers, their contents and relationships with other analytical data. Food Chem.

[15] Abegaz K, Beyene F, Langsrud T, Narvhus JA (2002). Indigenous processing methods and raw materials of borde, an Ethiopian traditional fermented beverages. J Food Technol Africa.

[16] Abawari RA (2013). Indigenous processing methods and raw materials of keribo: an Ethiopian traditional fermented beverage. J Food Resour Sci.

[17] Cocciardi RA, Ismail AA, Sedman J (2005). Investigation of the potential utility of single-bounce attenuated total reflectance Fourier transform infrared spectroscopy in the analysis of distilled liquors and wines. J Agric Food Chem.

[18] Dragone G, Mussatto SI, Oliveira JM, Teixeira JA (2009). Characterization of volatile compounds in an alcoholic beverage produced by whey fermentation. Food Chem.

[19] Savchuk SA, Vlasov VN, Appolonova SA, Arbuzov VN, Vedenin AN, Mezinov AB, Grigor’yan BR (2001). Application of chromatography and spectrometry to the authentication of alcoholic beverages. J Anal Chem.

[20] AOAC International (1990). Fifteenth ed., AOAC official method of analysis of wines.

[21] Brereton P, Hasnip S, Bertrand A, Wittkowski R, Guillou C (2003). Analytical methods for the determination of spirit drinks. Trends Anal Chem.

[22] Fletcher PJ, Van Staden JF (2003). Determination of ethanol in distilled liquors using sequential injection analysis with spectrophotometric detection. Anal Chim Acta.

[23] Debebe G (2006) Determination of ethanol level in beverages. Master Thesis, Addis Ababa University, Addis Ababa

[24] Yohannes T, Melak F, Siraj K (2013). Preparation and physicochemical analysis of some Ethiopian traditional alcoholic beverages. Afr J Food Sci.

[25] Berhanu A (2014). Microbial profile of *tella* and the role of gesho (*Rhamnus prinoides*) as bittering and antimicrobial agent in traditional *tella* (beer) production. Int Food Res J.

[26] Abbink J (1997). Competing practices of drinking and power: alcoholic “hegemonism” in Southern Ethiopia. Afr Stud Cent.

[27] Strunk DH, Hamman JW, Timmel BM (1979). Determination of proof of distilled alcoholic beverages, using an oscillating U-tube density meter. J AOAC.

[28] Mark FG, Vaughn TE (1980). Determination of proof of alcoholic beverages using oscillating U-tube density meter. J AOAC.

[29] Kovár J (1981). Oscillating U-tube density meter determination of alcoholic strength: analysis of paramter errors. J AOAC.

[30] Rebelein H (1995). Schnellmethode zur Bestimmung des Alkoholgehaltes in Likören und Branntweinen. Alkohol-Ind.

[31] Beutler H-O, Michal G (1977). Neue Methode zur enzymatischen Bestimmung von Äthanol in Lebensmitteln. Z Anal Chem.

[32] Pietsch H-P, Oehler R, Kasprick D (1968). Gaschromatographische Bestimmung von Äthanol in Spirituosen. Nahrung.

[33] Matthes D (1981). Alkoholbestimmungen mittels Dampfraum-Gaschromatographie—eine einfache, schnelle Methode für den Routinebetrieb. Branntweinwirtsch.

[34] Kovár J (1985). Determination of alcoholic strength in alcoholic beverages by gas-solid chromatography. J Chromatogr.

[35] Wang ML, Wang JT, Choong YM (2004). Simultaneous quantification of methanol and ethanol in alcoholic beverage using a rapid gas chromatographic method coupling with dual internal standards. Food Chem.

[36] Martin E, Iadaresta V, Giacometti JC, Vogel J (1986). Ethanol determination by HPLC in alcoholic beverages. Mitt Geb Lebensmittelunters Hyg.

[37] Buckee GK, Mundy AP (1993). Determination of ethanol in beer by gas chromatography (direct injection)-collaborative trial. J Inst Brew.

[38] Nordon A, Mills A, Burn RT, Cusick FM, Littlejohn D (2005). Comparison of non-invasive NIR and Raman spectrometries for determination of alcohol content of spirits. Anal Chim Acta.

[39] Garrigues JM, Pérez-Ponce A, Garrigues S, Guardia M (1997). Direct determination of ethanol and methanol in liquid samples by means of vapor phase-Fourier transform infrared spectrometry. Vib Spectrosc.

[40] Pérez-Ponce A, Rambla FJ, Garrigues JM, Garrigues S, Guardia M (1998). Partial least squares-Fourier transform infrared spectrometric determination of methanol and ethanol by vapour-phase generation. Analyst.

[41] Lachenmeier DW, Godelmann R, Steiner M, Ansay B, Weigel J, Krieg G (2010). Rapid and mobile determination of alcoholic strength in wine, beer and spirits using a flow-through infrared sensor. Chem Cent J.

[42] Patz C-D, Blieke A, Ristow R, Dietrich H (2004). Application of FT-MIR spectrometry in wine analysis. Anal Chim Acta.

[43] Coldea TE, Socaciu C, Fetea F, Ranga F, Pop RM, Florea M (2013). Rapid quantitative analysis of ethanol and prediction of methanol content in traditional fruit brandies from Romania, using FT-IR spectroscopy and chemometrics. Not Bot Horti Agrobo.

[44] Gallignani M, Garrigues S, Guardia M (1994). Derivative Fourier transform infrared spectrometric determination of ethanol in beers. Analyst.

[45] Lachenmeier DW (2007). Rapid quality control of spirit drinks and beer using multivariate data analysis of Fourier transform infrared spectra. Food Chem.

[46] Egidio VD, Sinelli N, Giovanelli G, Moles A, Casiraghi E (2010). NIR and MIR spectroscopy as rapid methods to monitor red wine fermentation. Eur Food Res Technol.

[47] Martelo-Vidal MJ, Vázquez M (2014). Evaluation of ultraviolet, visible, and near infrared spectroscopy for the analysis of wine compounds. Czech J Food Sci.

[48] Lehtonen P, Hurme R (1994). Liquid chromatographic determination of sugars in beer by evaporative light scattering detection. J Ints Brew.

[49] Yukihiro O, Fred MW, Alfred AC (2007). Near-infrared spectroscopy in food science and technology.

[50] Urtubia A, Pérez-Correa JR, Meurens M, Agosin E (2004). Monitoring large scale wine fermentations with infrared spectroscopy. Talanta.

[51] Nagarajan R, Gupta A, Mehrotra R, Bajaj MM (2006). Quantitative analysis of alcohol, sugar, and tartaric acid in alcoholic beverages using attenuated total reflectance spectroscopy. J Autom Methods Manag Chem.

[52] Arzberger U, Lachenmeier DW (2008). Fourier transform infrared spectroscopy with multivariate analysis as a novel method for characterizing alcoholic strength, density, and total dry extract in spirits and liqueurs. Food Anal Methods.

[53] Kolomiets OA, Lachenmeier DW, Hoffmann U, Siesler W (2010). Quantitative determination of quality parameters and authentication of vodka using near infrared spectroscopy. J Near Infrared Spectrosc.

[54] Fu Q, Wang J, Lin G, Suo H, Zhao C (2012). Short-wave near-infrared spectrometer for alcohol determination and temperature correction. J Anal Methods Chem.

[55] Friedel M, Patz C-D, Dietrich H (2013). Comparison of different measurement techniques and variable selection methods for FT-MIR in wine analysis. Food Chem.

[56] Shen F, Wu Q, Wei Y, Liu X (2016). Evaluation of near-infrared and mid-infrared spectroscopy for the determination of routine parameters in chinese rice wine. J Food Process Preserv.

[57] Sanagi MM, Ling SL, Nasir Z, Ibrahim WAW, Naim AA (2009). Comparison of signal-to-noise, blank determination, and linear regression methods for the estimation of detection and quantification limits for volatile organic compounds by gas chromatography. J AOAC Int.

[58] Bahiru B, Mehari T, Ashenafi M (2001). Chemical and nutritional properties of ‘*Tej*’, an indigenous Ethiopian honey wine: variations within and between production units. J Food Technol Africa.

[59] Guranda HA (2013) Alcohol use amongst psychiatric in-patients in a mental hospital in Ethiopia. Master Thesis, University of South Africa, Pretoria

